# A Characterization of the Humoral Immune Response to Human Endogenous Retroviruses and *Mycobacterium paratuberculosis* in Crohn’s Disease

**DOI:** 10.3390/pathogens14040361

**Published:** 2025-04-07

**Authors:** Alishba Fayyaz, Luigi Cugia, Marta Noli, Somaye Jasemi, Elena Rita Simula, Leonardo A. Sechi

**Affiliations:** 1 Department of Biomedical Sciences, University of Sassari, 07100 Sassari, Italy; a.fayyaz@student.unisi.it (A.F.); martanoli@outlook.it (M.N.); sjasemi@uniss.it (S.J.); 2 Department of Medical Biotechnologies, University of Siena, 53100 Siena, Italy; 3 Gastroenterology and Digestive Endoscopy Department, Azienda Ospedaliera Universitaria di Sassari, 07100 Sassari, Italy; luigi.cugia@aouss.it; 4 SC Microbiologia e Virologia, Azienda Ospedaliera Universitaria, 07100 Sassari, Italy

**Keywords:** Human Endogenous Retroviruses, Crohn’s patients, *Mycobacterium paratuberculosis*, humoral immune response

## Abstract

Crohn’s disease (CD) is a multifactorial polygenic inflammatory bowel disease linked to aberrant immune response. *Mycobacterium paratuberculosis* (MAP) has been associated with CD; however, detecting MAP in CD tissues remains highly challenging. Recently, Human Endogenous Retroviruses (HERVs) differential gene expression has been reported in CD, but little is known about the involvement of MAP and HERVs in CD pathology. This study aimed to characterize the humoral response against HERV-K, HERV-W, and MAP antigens using an indirect ELISA in plasma samples from CD patients and age- and gender-matched healthy controls (HCs). We observed a significant antibody response against HERV-K and HERV-W epitopes in CD patients in comparison to MAP epitopes, as well as a higher overall antibody response in patients compared to HCs. This study is the first to report the presence of humoral immune response against HERVs antigens in CD. Considering the pro-inflammatory nature of CD, HERVs may contribute to the development or progression of disease in genetically predisposed individuals. However, further research is needed to better understand the complex role of HERVs in CD.

## 1. Introduction

Crohn’s disease (CD) is an inflammatory disease of the digestive tract characterized by diarrhea, abdominal cramps, and in some cases, due to persistent inflammation, it may cause complicated symptoms like fistulation, stricture, and intestinal perforation [1]. CD is a multifactorial polygenic condition [2] often linked to aberrant immune response to gut microbial flora in genetically predisposed individuals [3].

Numerous studies have explored the cause of CD over the past 40 years. Some conclusive studies linked *Mycobacterium avium subspecies paratuberculosis* (MAP) with CD after identifying insertional sequences IS900 unique to MAP through PCR [4]. In addition to that, antibodies against MAP antigen hsp65 have also been detected in CD patient serum [5]. Additionally, Significant attention has been given to the association between MAP and CD in genetically predisposed individuals due to CD’s similar pathophysiology to Johne’s disease, a chronic gastroenteritis condition in ruminants [6]. While these studies are compelling, the role of MAP in CD remains debated due to challenges in isolating it from patient samples [7].

Human Endogenous Retroviruses (HERVs) are retrovirus-derived DNA sequences that accumulated in the genome over the past 100 million years through multiple integration events. When retroviruses infect germ cells, the HERV provirus integrates into the host genome and is subsequently transmitted across generations in a Mendelian inheritance pattern [8,9]. Over time, HERVs lost the ability to produce infectious progeny due to accumulated mutations. However, their retroviral architecture remains preserved, consisting of three key genes, Gag (group-associated antigens), Pol (polymerase), and Env (envelope), surrounded by 5′ and 3′ regulatory LTRs (long terminal repeats) [10].

HERVs play a critical role in physiological processes like stem cell pluripotency and placenta formation [11,12,13]. However, aberrant HERV expression has been implicated in a range of diseases, including neurological disorders, autoimmune diseases, cancers, and COVID-19 infection [14,15,16]. For instance, HERVs involvement has been linked to multiple sclerosis, amyotrophic lateral sclerosis, autism, Parkinson’s disease, and schizophrenia [15,17,18,19,20,21,22]. HERVs reactivation has also been observed in autoimmune conditions such as rheumatoid arthritis, type 1 diabetes, and celiac disease [14,23,24]. Additionally, HERV differential gene expression has been detected in leukemia, prostate cancer, breast cancer, and germ cell tumors, suggesting a role in tumor progression [25,26,27].

Factors like viral and bacterial infections, caffeine, smoking, immune factors, and gene mutations play a critical role in HERV activation [24,28,29,30,31,32,33,34,35] and can contribute to the etiology of various diseases. One particular example is Herpes Simplex Virus 1’s involvement in Parkinson’s disease, in which HERV might play a protective role [36,37]. A recent study also suggested that stress, such as stimulated microgravity, may also contribute to aberrant HERV expression [38,39]. HERV-related activities can contribute to the variable expression of HERV RNA, synthesis of retroviral-like components, novel promoters, and oncogene activation [40,41].

Studies have reported the possible link between MAP and CD. However, a definitive answer remains elusive, underscoring the need for further investigation. Likewise, limited information on the potential role of HERVs in the onset or progression of CD, as well as their possible link to MAP in Crohn’s pathology, is available. Therefore, this study explored the role of HERVs and MAP in CD by analyzing the serological levels of HERVs and MAP peptides to elucidate their potential contribution to CD pathogenesis in comparison to the general population.

## 2. Materials and Methods

### 2.1. Study Population

Crohn’s patients were diagnosed based on European guidelines for the diagnosis and management of CD [42]. For each CD patient, an age- and gender-matched healthy control (HC) was included in this study. Variable numbers of samples were tested for each HERV and MAP peptide due to differences in sample availability throughout this study (Table 1). Written informed consent was obtained from all participants after full disclosure of information in compliance with regional and national regulations. CD patients with comorbidities of autoimmune diseases and active infections were excluded from the studied cohort. All CD patients and HCs were residents of Sardinia, Italy.

### 2.2. Blood Samples

5 mL peripheral venous blood sample was collected in an EDTA vial from each subject, and plasma was separated within 12 h of collection using Histopaque^®^-1077 (Sigma-Aldrich, St. Louis, MO, USA). 500 µL plasma aliquots were stored at −20 °C for short-term (<6 months) and at −80 °C for long-term (>6 months) storage.

### 2.3. Peptides

Epitopes derived from HERV (HERV-K(19–37) and HERV-W(248–262)) and MAP (MAP 38-65c(125–138), MAP 1,4-α-gbp(157–173), and MAP 2404c(70–85)) were designed using Epitope Database (Bebipred Linear Epitope Prediction 2.0) and Analysis Resource (https://www.iedb.org) software (Table 2). These epitopes were selected based on bioinformatics predictions, prioritizing regions of high antigenicity to ensure optimal immune recognition. All peptides were synthesized at >95% purity (LifeTein, South Plainfield, NJ, USA) and later quantified using HPLC. These peptides were then re-suspended at 10 mM in DMSO and stored at −80 °C.

### 2.4. ELISA Assays

Indirect enzyme-linked immunosorbent assay was used to identify specific plasma antibodies (Abs) against selected antigens HERV-K_(19–37)_, HERV-W_(248–262)_, MAP 38-65c_(125–138)_, MAP 1,4-α-gbp_(157–173)_, and MAP 2404c_(70–85)_. First, 96-well Nunc-Immuno Plates (Nalgen Nunc International, Rochester, NY, USA) were coated with 10 µg/mL of each peptide dissolved in 0.05 M carbonate–bicarbonate buffer, pH 9.5 (Sigma, St. Louis, MO, USA), and incubated overnight at 4 °C. Plates were then washed with 0.05% Tween-20 Tris Buffered Saline (TBS-T). Plates were blocked for 1 h with 1% skim milk in 1X-TBS and later washed twice with 0.05% Tween-20 washing buffer. Subsequently, 5 µL of plasma sample was added to 95 µL of blocking buffer, incubated for 2 h at room temperature, then washed four times with TBS-T, followed by the addition of 100 µL of secondary Ab IgG for 1 h. The secondary Ab, alkaline phosphatase-conjugated goat anti-human IgG polyclonal Ab, was added to the plate at a 1:1000 dilution (Sigma). ELISA plates were washed again four times in TBS-T buffer. The substrate, p-Nitrophenyl Phosphate (Sigma) tablets, was dissolved in 20 mL milli-Q water. Plates were incubated at room temperature with 200 µL of substrate for 15 min. The reaction was then stopped using 50 µL of 3N NaOH stop solution. Absorbance was recorded at the 405 nm wavelength using a SpectraMax Plus 384 microplate reader (Molecular Devices, Sunnyvale, CA, USA). Data were normalized to minimize experimental variation based on the reactivity of the positive control (absorbance reactivity set at 1.0 arbitrary unit) included in each plate. Immobilized peptide coated with IgG without a plasma sample was used as a negative control to minimize background noise.

### 2.5. Statistical Analysis

Data were analyzed with GraphPad Prism 8.0 (GraphPad Software Inc., La Jolla, CA, USA). The Shapiro–Wilk test was used to assess whether the sample distribution followed the Gaussian distribution. As samples were not normally distributed, the Mann–Whitney test was applied to analyze non-parametric data. ROC analysis was used to calculate the cut-off value for positivity in each dataset with specificity ≥90%, and sensitivity selected accordingly. Fisher’s exact test was used to determine statistically significant differences between CD patients and HCs groups. A *p*-value less than 0.05 was considered statistically significant. The Spearman test was used to determine the correlation between the tested HERV and MAP peptides.

## 3. Results

### 3.1. Antibody Response to HERV and MAP Immunogenic Epitopes in Crohn’s Disease Patients Versus Healthy Controls

**Figure 1:**
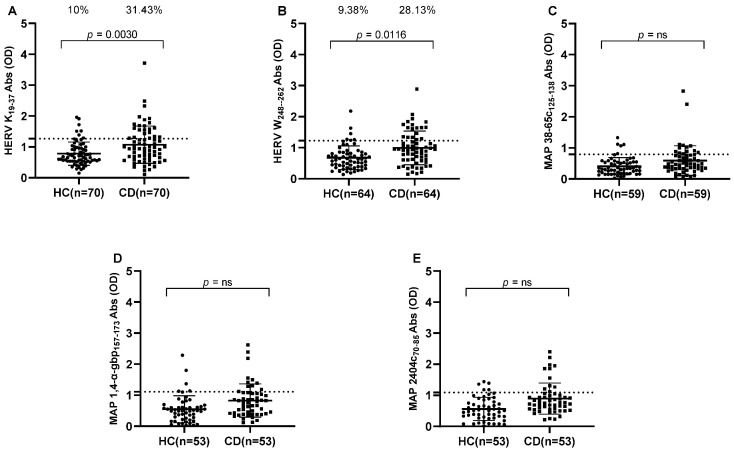
ELISA-based antibodies reactivity analysis against HERV and MAP peptides in CD patients and HCs. Plasma samples from subjects were tested against HERV-K_(19–37)_ (**A**), HERV-W_(248–262)_ (**B**), MAP 38-65c_(125–138)_ (**C**), MAP 1,4-α-gbp_(157–173)_ (**D**), and MAP 2404c_(70–85)_ (**E**). Dashed lines indicate thresholds for Abs calculated by ROC analysis, which were used to assess sample positivity. *p*-values derived from Fisher’s exact test and AUC are indicated in the graph, along with percentages of positive patients in the upper section of each graph. A *p*-value greater than 0.05 was considered statistically significant, while “ns” indicates non-significant results.

Our study included CD patients and their age-matched HCs (Table 1). Abs were detected in the plasma samples of CD patients and HCs against HERV-K, HERV-W, MAP 38-65c, Map 1,4-α-gbp, and MAP 2404c (Figure 1). Our patient samples also showed increased levels of positivity against HERV peptides in comparison to MAP peptides.

HERV-K showed the highest immunogenicity, of 31.43% (*n* = 22) for CD patients and 10% (*n* = 7) for HCs (cut-off value = 1.27, AUC = 0.6553, *p* = 0.0030) (Figure 1A). Likewise, Abs against HERV-W displayed high plasma positivity, accounting for 28.13% (*n* = 18) in CD patients and 9.38% (*n* = 6) in HCs (cut-off value = 1.225, AUC = 0.6881, *p* = 0.0116) (Figure 1B).

All three MAP peptides, MAP 38-65c, MAP 1,4-α-gbp, and MAP 2404c, showed significantly different immunogenicity in CD patients and HCs (Mann–Whitney *p*-values = 0.0097, 0.0021, and 0.0002, respectively), but positive plasma reactivity at selected ≥ 90% sensitivity cut-offs among all MAP peptides tested in CD patients and HCs was not significant according to the Fisher test (Figure 1C–E).

**Figure 2:**
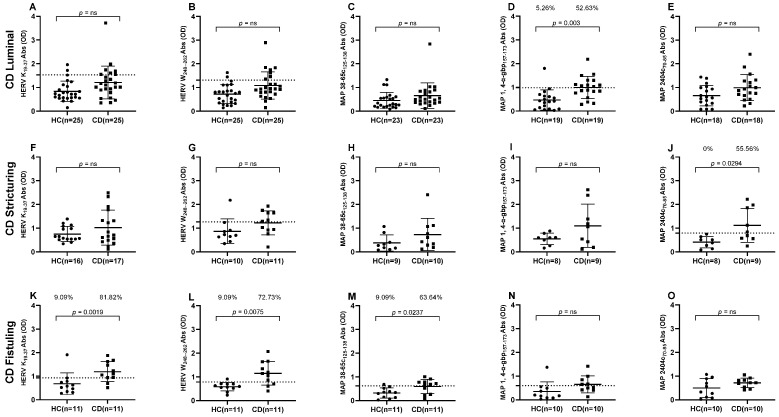
ELISA based antibodies reactivity analysis against HERV and MAP peptides in CD patients and HCs, categorized by CD clinical symptoms. (**A**–**E**) Healthy and luminal CD subjects tested against HERV-K_(19–37)_, HERV-W_(248–262)_, MAP 38-65C_(125–138)_, MAP 1,4-α-gbp_(157–173)_, and MAP 2404c_(70–85)_ peptides, respectively. (**F**–**J**) Healthy and stricturing CD subjects tested against HERV-K_(19–37)_, HERV-W_(248–262)_, MAP 38-65C_(125–138)_, MAP 1,4-α-gbp_(157–173)_, and MAP 2404c_(70–85)_ peptides, respectively. (**K**–**O**) Healthy and fistuling CD subjects tested against HERV-K_(19–37)_, HERV-W_(248–262)_, MAP 38-65C_(125–138)_, MAP 1,4-α-gbp_(157–173)_, and MAP 2404c_(70–85)_ peptides, respectively. Dashed lines indicate Abs thresholds calculated by ROC analysis, which were used to assess sample positivity. *p*-values derived from Fisher’s exact test along with percentages of positive patients are displayed in upper sections of graphs. A *p*-value greater than 0.05 was considered statistically significant, while “ns” indicates non-significant results.

We then stratified available data based on the clinical manifestation of CD into three distinct phenotypes: luminal CD, stricturing CD, and fistuling CD. This categorization allowed us to observe significant variations in the prevalence of plasma Abs against MAP and HERV peptides across different Crohn’s phenotypes. Results indicate an intriguing pattern of higher Abs against the HERV-K peptide in fistuling CD group that accounted for 81.82% (*n* = 9) in comparison to 9.09% (*n* = 1) in positive HCs (cut-off = 0.93, AUC= 0.8347, *p* = 0.0019) (Figure 2K), while no significant differences in HERV-K Abs levels were observed in luminal CD and stricturing CD groups (Figure 2A,F). Likewise, when HERV-W Abs were analyzed in distinct CD subgroups in comparison to HCs, higher Abs levels were found against fistuling CD in comparison to luminal CD and stricturing CD groups, in which no significant differences in Abs prevalence were found against healthy subjects. A 72.73% (*n* = 8) reactivity was determined against HERV-W in the fistuling CD group versus 9.09% (*n* = 1) in HCs (cut-off = 0.785, AUC = 0.8512, *p* = 0.0075) (Figure 2L).

Our analysis revealed particularly interesting results for MAP peptides, indicating that MAP 38-65c had higher significant immunogenicity, 63.64% (*n* = 7), in the fistuling CD group in comparison to 9% (*n* = 1) in healthy individuals (cut-off = 0.62, AUC = 0.77, *p* = 0.023) (Figure 2M). MAP 1,4-α-gbp was the only peptide that accounted for a significant Abs presence in the luminal CD group, 52.63% (*n* = 10), in comparison to 5.26% (*n* = 1) in HCs (cut-off = 0.985, AUC = 0.831, *p* = 0.003) (Figure 2D). Likewise, a higher Abs prevalence was reported against MAP 2404c in the stricturing CD group, which was 55.56% (*n* = 5) in comparison to 0% for HCs (cut-off = 0.795, AUC = 0.8472, *p* = 0.0294) (Figure 2J).

### 3.2. Correlation Analyses of HERV and MAP Antibodies

**Figure 3:**
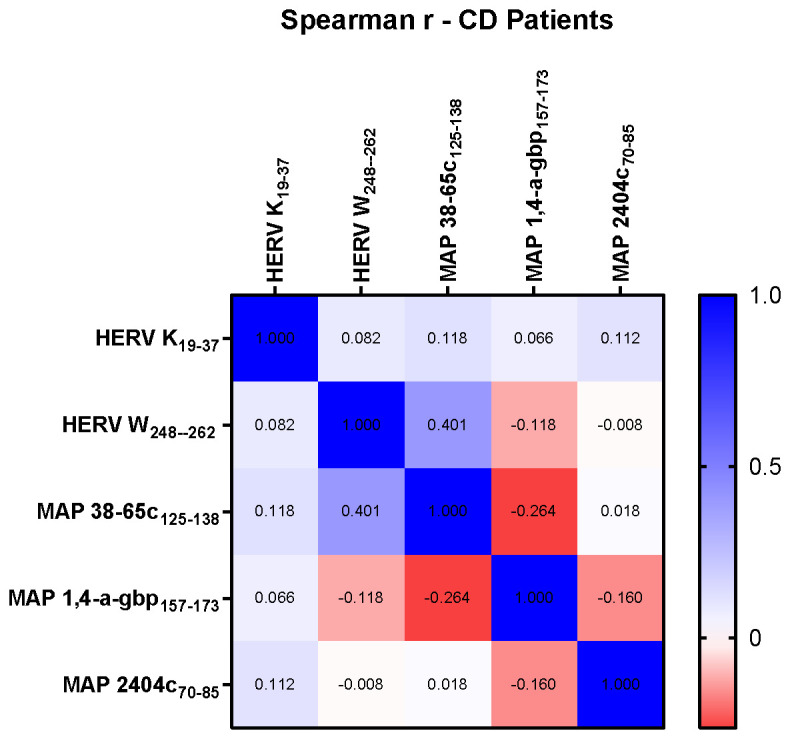
Heat-map indicating r values computed using Spearman correlation test among peptides HERV-K_(19–37)_, HERV-W_(248–262)_, MAP 38-65C_(125–138)_, MAP 1,4-α-gbp_(157–173)_, and MAP 2404c_(70–85)_.

We performed correlation analysis of plasma Abs levels using the Spearman correlation test for non-parametric data to determine the association between the antigenic potential of tested HERV and MAP peptides. We found evidence of strong correlations in some tested peptides, but not in all, both in the collective cohort of CD samples and in subcategorized CD data based on CD phenotype. CD patient samples accounted for the highest r coefficient of 0.4 for the HERV-W and MAP 38-65c pairwise analysis (*p* = 0.002). No significant correlation was found in CD samples for other peptides pairwise analysis (Figure 3).

**Figure 4:**
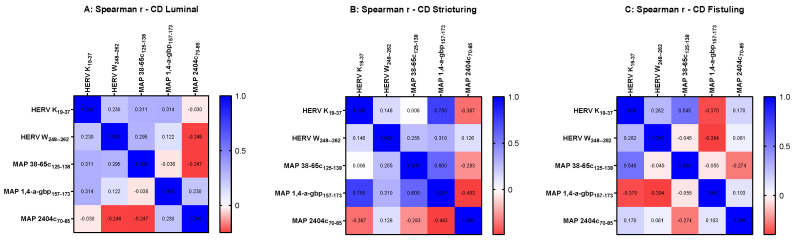
Heat-map indicating r values computed using Spearman correlation analysis among peptides HERV-K_(19–37)_, HERV-W_(248–262)_, MAP 38-65C_(125–138)_, MAP 1,4-α-gbp_(157–173)_, and MAP 2404c_(70–85)_ in Crohn’s disease cohort categorized based on disease behavior as luminal (**A**), stricturing (**B**), and fistuling (**C**).

However, when we analyzed association by categorizing data based on the distinct behavior of CD, our results revealed an intriguing pattern of HERV and MAP peptides suggesting a potential link for their expression in the manifestation of symptoms of CD. An r value equal to 0.75 was obtained in MAP 1,4-α-gbp/HERV-K pairwise analysis (*p* = 0.025) in the stricturing CD group (Figure 4B), indicating a strong connection between HERV-K and MAP 1,4-α-gbp contributing to developing strictures in Crohn’s patients.

## 4. Discussion

CD is an autoimmune inflammatory disorder of the gastrointestinal tract, particularly affecting the ileum and colon. CD cases are globally rising in adults and pediatrics associated with complex symptoms like abscessed strictures and fistulas [43]. Multiple factors, like genetics and environment, can contribute to the onset and progression of etiology. However, one definite causative agent for CD has not been defined, but some studies highlight MAP as a potential triggering factor for the onset of Crohn’s symptoms due to its similarities with Johne’s disease, which is an infectious pathology of the small intestine in ruminants caused by MAP [44,45].

Studies have reported higher transcriptional levels of various HERV genes like HERV-H pol and HERV-K pol in inflammatory bowel diseases (IBDs) like Crohn’s and ulcerative colitis [46]. HERV-W envelope protein (synctin 1) has been shown to upregulate C-reactive protein (CRP) expression, which is a well-recognized biomarker of inflammation in inflammatory bowel disease [47,48]. Differential expression of HERVs like HML, HERV-ERI, HERV-W, and HERV-H has also been observed in a tissue-dependent manner in the ileal and colon of CD patients. HERV envelope genes (synctin 1 and 2) have shown variable expression in inflamed and non-inflamed tissues in CD patients, suggesting that their expression might modify immune response in the gut barrier [49]. Our study supports the involvement of HERVs in the pathogenesis of CD.

A possible mechanism by which HERVs could contribute to CD might be through disrupting the immune homeostasis of TNF-α, the cGAS/STING/NF-Κb pathway, and M1 macrophages. Tumor necrosis factor (TNF-α) plays a crucial role in the immune-mediated response by promoting immune cell activation and wound healing. Its level can be upregulated by HERV-W env [50]. Anti-TNF therapies like infliximab and adalimumab are commonly used to treat CD by reducing inflammation [51,52,53]. The cGAS/STING/NF-Κb inflammatory cascade and M1 macrophages are involved in maintaining homeostasis in colon mucosa, which can be interfered with by HERVs. If retroviruses are inhibited with a retrovirus reverse transcriptase inhibitor azidothymidine, this can eliminate inflammatory bowel disease (colitis) symptoms in mice [54].

Unfortunately, we did not find a statistically significant reactivity difference between patients and HCs with the Fisher’s test against MAP antigens (many were closer to significance), but similar results have been previously reported as well [7,55]. However, when we analyzed these stratified data based on CD’s distinct behavior, particularly focusing on luminal, stricturing, and fistuling CD phenotypes, an interesting observation was made regarding the prevalence of MAP and HERVs peptides across different disease behaviors. These results revealed intriguing patterns, indicating the presence of Abs against various MAP antigens in different phenotypes of CD, and as the severity of disease progressed from luminal and strictures to fistulation we observed a significantly higher seropositivity rate for HERVs antigens in CD patients in comparison to HCs. We initially hypothesized the involvement of MAP in transactivation of HERVs, but we could not observe any strong correlation between HERVs and MAP peptides; interestingly we identified a strong association between MAP 1,4-α-gbp and HERV-K in the stricturing CD group, suggesting a potential link between microbial or viral peptide expression and the manifestation of particular CD symptoms.

The assumption of MAP involvement in the transactivation of HERVs and its possible role in triggering the pathophysiology of CD are supported by elevated CD68 expression in macrophages-activated granulomatous lesions in infected goats. However, the humoral response of CD patients indicates that the involvement of MAP in the pathogenesis of CD is a more complex cascade than we anticipated [56,57].

Despite valuable insights, our study had some limitations. The sample sizes of CD patients tested for each peptide varied because some samples were exhausted and no longer available. The sample size of stratified subgroups under investigation was relatively small, and therefore prompts the need for the validation of our results with a larger cohort. Although our study highlights the potential involvement of HERVs in the immune response against CD, the current experimental design does not identify the molecular pathways involved in HERV activation and its involvement in the pathophysiology of CD, leaving room for further investigation. Similarly, our experimental strategy needs to be refined to determine whether MAP contributes to CD and, if so, how, to elucidate its mechanism of action. However, to our knowledge this is the first study that reports the presence of an immune response against HERV antigens in CD patients.

In conclusion, while our findings suggest a potential role of HERVs in the development of CD, further research is needed to fully elucidate the mechanisms of HERV activation and their potential as biomarkers for CD. To address these questions, our future studies will integrate gene expression profiling and validation using qPCR to provide a comprehensive understanding of the contribution of HERVs in CD pathogenesis. These efforts will be critical in advancing our knowledge and could ultimately lead to improved diagnostic and therapeutic strategies for CD.

## Figures and Tables

**Table 1:** Demographics of Crohn’s disease patients and healthy controls tested against each peptide.

Peptides	CD				HC			
	Samples (*n*)	Median Age	M	F	Samples (*n*)	Median Age	M	F
HERV-K	70	40.5	36	34	70	42	37	33
HERV-W	64	40	35	29	64	41	35	29
MAP 38-65c	59	41	31	28	59	43	32	27
MAP 1,4-α-gbp	53	39	25	28	53	39	26	27
MAP 2404c	53	39	25	28	53	39	26	27

**Table 2:** Epitopes identified in MAP and HERV.

Epitopes	Amino Acid Sequence	Position
HERV-K	VWVPGPTDDRCPAKPEEEG	19–37
HERV-W	NSQCIRWVTPPTQIV	248–262
MAP 38-65c	MIAVALAGLAANFV	125–138
MAP 1,4-α-gbp	GTVELLGGPLAHPFQPL	157–173
MAP 2404c	RGFFYTPKTRREAEDL	70–85

## Data Availability

Data are contained within the article.
